# Comparison of cytomorphology and histomorphology in myelodysplastic syndromes

**DOI:** 10.3389/fonc.2024.1359115

**Published:** 2024-04-11

**Authors:** Kathrin Nachtkamp, Corinna Strupp, Rosa Faoro, Norbert Gattermann, Sascha Dietrich, Ulrich Germing, Stefan Baldus

**Affiliations:** ^1^ Department of Hematology, Oncology and Clinical Immunology, Heinrich-Heine-University, Düsseldorf, Germany; ^2^ Department of Pathology, Heinrich-Heine-University, Düsseldorf, Germany

**Keywords:** myelodysplastic syndromes, cytomorphology, histopathology, prognosis, WHO2022

## Abstract

Gold standard for the establishment of the diagnosis of myelodysplastic syndromes (MDS) are cytomorphological features of hematopoietic cells in peripheral blood and bone marrow aspirates. There is increasing evidence that bone marrow histomorphology not only aids in the diagnosis of MDS but can provide additional prognostic information, particularly through assessment of fibrosis and cellularity. However, there is only sparse data on direct comparison between histological and cytomorphological findings within the same MDS patient cohort. Therefore, we performed such an analysis under exceptionally well-standardized conditions. We reexamined biopsy material of 128 patients from the Düsseldorf MDS registry who underwent bone marrow trephine biopsy (in addition to bone marrow aspiration) at the time of diagnosis, addressing the following items: a. Analysis of concordance of diagnoses made by histology and cytomorphology b. Analysis of additional information by histology with regard to the diagnosis and prognosis. The respective biomaterials were available at our institution and had been processed according to unchanged protocols between 1992 and 2010. Fresh histopathological sections were obtained from the tissue blocks, stained under identical conditions and re-assessed by a designated expert pathologist (C.B.) without knowledge of the previous histopathological report or the respective cytomorphological diagnosis. The latter, likewise, was uniformly made by the same expert cytomorphologist (U.G.). Histopathology of bone marrow trephine biopsies reliably captured the diagnosis of MDS. Assignment to the diagnostic WHO subgroup was not entirely concordant with cytomorphology, mainly due to incongruences between the proportion of CD34-positive cells on histopathology and the cytomorphological blast count. Histopathology provided additional diagnostic and prognostic information with high diagnostic and prognostic significance, such as fibrosis. Likewise, histopathology allowed more reliable estimation of bone marrow cellularity.

## Introduction

Myelodysplastic syndromes are a heterogeneous group of clonal stem cell disorders, morphologically defined by dysplastic features of hematopoietic cells and increasing impairment of hematopoietic cell differentiation, recognizable by an elevated proportion of bone marrow blasts in higher-risk MDS (1–7). Disturbed maturation entails functional defects of blood cells, as well as peripheral blood cytopenias (7, 8). MDS also carries the risk of transformation into acute myeloid leukemia (9). Primary myelodysplastic syndromes, which account for about 90% of MDS cases, lack an apparent cause, whereas radiotherapy or noxious agents, such as chemotherapy or organic solvents, are present in the medical history of patients with secondary or therapy related MDS. In rare cases, a familial predisposition to clonal hematopoietic disorders is recognized.

Myelodysplastic syndromes are categorized by considering dysplastic features, blast count, and cytopenias. The diagnosis is usually established by cytomorphology. Despite peripheral blood cytopenia, the bone marrow in MDS is generally hyper- or normocellular.

Since the 1980s, several classifications have been established that separate morphologically discernible, prognostically relevant types of MDS. The FAB classification, published in 1982, was solely based on morphological criteria (10). The WHO classification, first published in 2001, refined the MDS subtypes and was the first to require chromosomal analyses and to include a chromosomal aberration (del5q) as a disease-defining marker. Revised versions were published in 2016 and 2022 (11–14).

While diagnostic criteria for MDS mainly rely on cytomorphological features, some MDS patients may show bone marrow fibrosis, which can only be assessed on histopathology. In addition, histopathology is deemed superior to cytomorphology regarding assessment of bone marrow cellularity.

Our analysis compares cytomorphological and histopathological findings and their degree of conformity in a cohort of MDS cases with respect to diagnostic accuracy and prognostic significance that is unique in terms of uniform preparation of diagnostic samples and lack of interobserver variability.

## Materials and methods

We analyzed bone marrow aspirates of 152 MDS patients diagnosed between 1992 and 2010 by central cytomorphology according to the WHO classification (12, 13). Bone marrow trephine biopsies obtained at the time of diagnosis were also available from these patients. Data regarding clinical features, cell counts, and the course of disease were obtained from the Duesseldorf MDS registry. We considered date of birth, gender, time of diagnosis, WHO/FAB subtype, treatment history, and outcome. Data closure/end of follow-up was July 1^st^, 2012, or date of death. Only a small subset of patients was lost to follow-up. The availability of the abovementioned data set was mandatory for inclusion in the analysis. 128 patients were assessable for further analysis.

The bone marrow trephine biopsy taken at the time of diagnosis was used for preparing new histopathological sections and stains, which were assessed by a single reviewing pathologist (SB) who had no knowledge of the cytomorphological evaluation, apart from the information “patient with myelodysplastic syndrome”. Bone marrow biopsies we carried out and processed according to local standards. Immunohistochemical staining with an anti-CD34 antibody was used to visualize immature hematopoietic progenitor cells. Histological slides were routinely stained with hematoxylin-eosin (HE), periodic acid Schiff reagent (PAS), Giemsa, silver impregnation according to Gomori, and iron staining (Berliner-Blau). In addition, the naphthol AS-D chloroacetate esterase reaction was used to highlight neutrophilic granulopoiesis.

The following morphological parameters were assessed by standardized procedures and were evaluated semi quantitatively (**Table 1**): cellularity of the specimen in comparison to an age-related control cohort, maturation and dysplasia of megakaryopoiesis, cellularity of erythropoiesis and proportion of erythroid cells relative to granulopoietic cells, degree of fibrosis, bone marrow iron content, and percentage of CD34-positive cells in relation to the overall cellularity of the bone marrow. Length, quality, and number of evaluable intertrabecular areas were also assessed.

**Table 1:** Morphologic parameters.

morphological parameters assessed by histopathology	
bone marrow cellularity	<40% (hypocellular)
	40-60% (normocellular)
	>60% (hypercellular)
proportion of adipocytes	0-10%
	11-30%
	31-50%
	51-70%
	>70%
percentage of erythropoiesis	0-20%
	21-40%
	41-60%
	61-80%
	>80%
degree of dysplasia of megakaryopoiesis	0= no signs of dysplasia
	1= low to moderate
	2=marked signs of dysplasia
	3=high degree of dysplasia
bone marrow fibrosis	0= no fibrosis
	1= low degree
	2= high degree
	3= very high degree
iron content of reticulum cells	0= no iron/depletion
	1= low to normal amount
	2= increased amount
	3= markedly increased amount
percentage of CD34-positive cells (in relation to total cell count)	<1%
	1-2%
	3-5%
	6-10%
	11-15%
	16-20%
	21-30%
morphological parameters assessed by cytology	
bone marrow cellularity	hypocellular vs normocellular vs hypercellular
Dyserythropoiesis in marrow	percentage of erythropoiesis
	megaloblastoid changes
	multinuclearity
	nuclear budding
	nuclear bridges
	atypical mitoses
	sideroblastosis
	percentage of ringsideroblasts
	PAS positive red precursors
Dysmegakaryopoiesis in marrow	micromegakaryocytes
	mononuclear megakaryocytes
	hypersegmentes megakaryocytes
	multinuclearity of megakaryocytes
Dysgranulopoiesis in marrow	hyperplasia of granulopoiesis
	left shift of granulopoiesis
	medullary blast count
	Auer rods or Auer bodies (POX-staining)
	hypo/degranulation of white precursors
	pseudo-Pelger cells
	nuclear anomalies of granulocytes (hypersegmentation)
	deficiency of myeloperoxidase
	percentage of monocytopoiesis (esterase staining)
Other features	Percentage of lymphoid cells
	percentage of plasma cells
	iron storage

The correlating cytomorphological findings were taken from the Duesseldorf MDS registry. Cytomorphological assessment was performed according to standard operating procedures as reported by Germing et al. (**Table 1**) (15). Of note, 20 patients with the diagnosis of RAEB-T according to the FAB classification were included in the analysis.

To ensure homogeneity and comparability, histopathological and cytomorphological diagnoses were always established by the same reviewers, respectively (UG for cytomorphology, SB for histopathology). Statistical analyses were performed using SPSS. Comparison of blast count by cytomorphology versus histology was analyzed by nonparametrical Wilcoxon T-Test. Categorical variables were analyzed using Chi-Square-Test. All procedures were in accordance with the current version of the Helsinki Declaration. Informed consent was obtained from all patients included in the study.

## Results

### Patient characteristics

Of the 128 patients evaluable, 79.7% had deceased at the time of this analysis, 19.5% were documented alive at the time of data closure and 0.8% were lost to follow up.

Baseline characteristics of the cohort are presented in **Table 2a** and **Table 2b**. Patients’ MDS subtype according to both WHO 2016 and WHO 2022 classification are shown in **Table 2a**, **Table 2b** demonstrates the redistribution of patients from the WHO 2016 to 2022 classification. Median age was 67 years. Median survival time was 16.3 months after diagnosis (0-164.4 months).

**Table 2a:** Patients´ characteristics according to WHO 2016 and WHO 2022.

	n	
median age (range)	128	67 (23-90)
	n	percent
male	83	65%
female	45	35%
WHO2016		**n=128**
MDS-SLD	12	9.40%
MDS-RS-SLD	3	2.30%
MDS-MLD	18	14.10%
MDS-RS-MLD	7	5.50%
MDS-EB-1	15	11.70%
MDS-EB-2	25	19.50%
AML-MRC (RAEB-T)	18	14.10%
CMML 0,I	21	16.40%
CMML II	9	7.00%
IPSS		**n=89**
low	11	12.40%
intermediate-1	25	28.10%
intermediate-2	26	29.20%
high	27	30.30%
	n	
median age (range)	128	67 (23-90)
	n	percent
male	83	65%
female	45	35%
WHO2022		**n=128**
MDS-LB-SLD	10	7.80%
MDS-LB-MLD	10	7.80%
MDS-SF3B1	10	7.80%
MDS-IB1	13	10.20%
MDS-IB2	20	15.60%
MDS-F	7	5.50%
MDS-hypo	10	7.80%
AML-MRC	18	14.10%
CMML I	21	16.40%
CMML II	9	7.00%

**Table 2b:** Comparison of patients’ distribution between WHO 2016 and WHO 2022.

			WHO 2022										Total
			LB-SLD	LB-MLD	LB-SF3B1	IB1	IB2	MDS-F	MDS hypo	AML-MRC	CMML I	CMML II	
WHO 2016	MDS-SLD	n	10	0	0	0	0	0	2	0	0	0	12
		% who2016	83,3%	0,0%	0,0%	0,0%	0,0%	0,0%	16,7%	0,0%	0,0%	0,0%	100,0%
	MDS-RS-SLD	n	0	0	3	0	0	0	0	0	0	0	3
		% who2016	0,0%	0,0%	100,0%	0,0%	0,0%	0,0%	0,0%	0,0%	0,0%	0,0%	100,0%
	MDS-MLD	n	0	10	0	0	0	0	8	0	0	0	18
		% who2016	0,0%	55,6%	0,0%	0,0%	0,0%	0,0%	44,4%	0,0%	0,0%	0,0%	100,0%
	MDS-RS-MLD	n	0	0	7	0	0	0	0	0	0	0	7
		% who2016	0,0%	0,0%	100,0%	0,0%	0,0%	0,0%	0,0%	0,0%	0,0%	0,0%	100,0%
	MDS-EB-1	n	0	0	0	13	0	2	0	0	0	0	15
		% who2016	0,0%	0,0%	0,0%	86,7%	0,0%	13,3%	0,0%	0,0%	0,0%	0,0%	100,0%
	MDS-EB-2	n	0	0	0	0	20	5	0	0	0	0	25
		% who2016	0,0%	0,0%	0,0%	0,0%	80,0%	20,0%	0,0%	0,0%	0,0%	0,0%	100,0%
	AML-MRC (RAEB-T)	n	0	0	0	0	0	0	0	18	0	0	18
		% who2016	0,0%	0,0%	0,0%	0,0%	0,0%	0,0%	0,0%	100,0%	0,0%	0,0%	100,0%
	CMML 0,I	n	0	0	0	0	0	0	0	0	21	0	21
		% who2016	0,0%	0,0%	0,0%	0,0%	0,0%	0,0%	0,0%	0,0%	100,0%	0,0%	100,0%
	CMML II	n	0	0	0	0	0	0	0	0	0	9	9
		% who2016	0,0%	0,0%	0,0%	0,0%	0,0%	0,0%	0,0%	0,0%	0,0%	100,0%	100,0%
Total	n	10	10	10	13	20	7	10	18	21	9	128

Red marked numbers indicate the either most statistically relevant or most strinkingly differing parameters within the comparison of histo- and cytomorphology.

MDS diagnoses were made according to the WHO classification of 2016. 12 patients were diagnosed as MDS-SLD (9.4%), 25 patients as MDS-MLD with or without ring sideroblasts (19.6%), 3 patients (2%) as RARS, 15 patients (11.7%) as RAEB I, and 23 patients (18.0%) as RAEB II. In addition, 21 patients were diagnosed with CMML 0/I (16.4%), and 9 patients (7.0%) with CMML II. 20 patients (15.6%) belonged to the category of AML with myelodysplasia-related changes (the former RAEB-T diagnosis), with 20-30% medullary blasts.

### Histopathological analysis

Determinants of the interpretability of histopathological specimens are length of the trephine biopsy, number of evaluable intertrabecular areas, and overall quality of the sample. 53.9% of the trephine biopsies had a length between 0.6 and 1.0 cm, 26.6% had a length of >1.0 cm. Evaluability was assessed by subjective rating. 93% of all specimens were rated at least satisfactory (grade 3 of 6) and were thus evaluated for all parameters. In 9 cases (7.0%) the number of evaluable intertrabecular areas was less than 5. In 93% of all cases, 5-15 evaluable intertrabecular areas could be analyzed.

Median cellularity was 65%. 23.4% of all cases were hypocellular (e.g., bone marrow cellularity <40%), 21.1% normocellular (40-60% bone marrow cellularity), and 55.5% hypercellular (>60%).

### Histological versus cytomorphological findings

#### Medullary blast count/CD34+ cells

In direct comparison, the medullary blast count was underestimated by histopathology regardless of the proportion of blasts seen by cytomorphology (**Table 3**, p=0.001). For instance, in patients with a cytomorphologically assessed blast count of more than 20% (RAEB-T by definition), histopathology identified less than 5% blasts in 22.7% of these cases. The same was true for a cytomorphological blast count of 10-19%, where histopathology found a normal blast count (<5%) in 43%, and a blast count of 5-9% in 29% of these cases.

**Table 3:** Comparison of blast percentages assessed by cytomorphology with blast percentage assessed by staining of CD34 by histopathology (p<0.001).

		CD34+ cells by histopathology			
Blast count by cytology					
	**0-4%**	**5-9%**	**10-19%**	**20-29%**	**total**
**0-4%**	32	15	2	1	50
**5-9%**	14	9	4	1	28
**10-19%**	12	8	5	3	28
**20-29%**	5	4	6	7	22
					128

Red marked numbers indicate the either most statistically relevant or most strinkingly differing parameters within the comparison of histo- and cytomorphology.

Patients with a hypocellular marrow according to histopathology were more likely to present with a cytomorphologically assessed blast count below 5%, whereas hypercellularity correlated with blast counts above 10% (60.7% of patients with 10-19% blasts and 63.6% with ≥20% blasts, respectively). Nevertheless, 46.9% of patients with <5% medullary blasts presented with hypercellular marrow when diagnosed. The correlations did not reach statistical significance in our analyses (p=0.767).

#### Dysplastic features of megakaryopoiesis

Comparing dysmegakaryopoiesis according to histopathology and cytomorphology, there were 44 cases where megakaryopoiesis appeared inconspicuous on cytomorphology but showed at least mild to moderate signs of dysplasia on histopathology (**Table 4**, p=0.009). Conversely, among 9 cases that appeared normal on histopathology, 8 showed signs of dysplastic megakaryopoiesis on cytomorphological assessment.

**Table 4:** Comparison of assessment of dysmegakaryopoiesis (histology vs cytology) (χ^2 = ^17.0, p=0.009), dysmegakaryopoiesis assessed by histology vs cellularity by histology (χ^2 = ^17.0, p=0.009) and dysmegakaryopoiesis assessed by histology vs degree of myelofibrosis (χ^2 = ^33.2, p<0.00005).

		Dysplasia by histology			
Dysplasia by cytology					
	**No**	**Low-moderate**	**Marked signs**	**High degree of dysplasia**	**total**
**No**	1	21	17	6	45
**yes**	8	36	30	2	76
					121
Cellularity by histology					
	**No**	**Low-moderate**	**Marked signs**	**High degree of dysplasia**	**total**
**<40%**	6	18	5	1	30
**40-60%**	1	14	11	1	27
**>60%**	3	27	34	7	71
					128
Degree of myelofibrosis					
	**No**	**Low-moderate**	**Marked signs**	**High degree of dysplasia**	**total**
**No fibrosis**	5	11	4	0	20
**Grade 1**	5	40	39	4	88
**Grade 2**	0	8	7	4	19
**Grade 3**	0	0	0	1	1
					127

Red marked numbers indicate the either most statistically relevant or most strinkingly differing parameters within the comparison of histo- and cytomorphology.

Histopathological assessment of cellularity showed a positive correlation with the degree of dysmegakaryopoiesis. Hypercellular marrow was associated with a greater degree of dysplastic features (**Table 4**, p=0.009). More pronounced signs of dysmegakaryopoiesis, as assessed by histopathology, were also found in higher-risk MDS subtypes according to WHO 2016 that are characterized by elevated blast count as well as greater cellularity.

A high level of dysmegakaryopoiesis was less common in patients with a high degree of fibrosis (**Table 4**, p<0.00005). This may be due to an increased blast count and less residual normal hematopoiesis, both contributing to a diminished number of assessable megakaryocytes.

#### Cellularity

Histopathology is the gold standard for assessing bone marrow cellularity. When compared to the histopathology report, cytomorphology tends to overestimate cellularity (**Table 5**). A hypocellular marrow was diagnosed in 24.4% of cases by histopathology. Within that group, cytomorphology described a normocellular marrow in 44.8%, and even a hypercellular marrow in 48.3% of cases. Normocellularity was generally congruent when the finding of a normocellular marrow on histopathology was taken as the gold standard. Regarding hypercellularity, almost half of the cases diagnosed as hypercellular on histopathology were characterized as normocellular by cytomorphological assessment.

**Table 5:** Cellularity assessed by histology vs cytology (χ^2 = ^4.33, p=0.36).

		Histology		
Cytology				
	**<40%**	**40-60%**	**>60%**	**total**
**hypocellular**	2 (18%)	3 (27%)	6 (55%)	11
**normocellular**	13 (24.5%)	16 (30.2%)	24 (45.3%)	53
**hypercellular**	14 (25.5%)	8 (14.5%)	33 (60%)	55

Considering histopathological cellularity in relation to the WHO2016 subgroups, there was hypercellularity in MDS types with increased blast count such as MDS-EB1 (46.7% of cases with MDS-EB1) and MDS-EB2 (40%), and in CMML with (88.9%) or without (85.7%) increased blast count (p<0.01). Inversely, the incidence of hypocellularity decreased in the aforementioned subgroups. In low-blast WHO subtypes the distribution of cellularities was as follows: hypocellular 46,7%, normocellular 40,7%, hypercellular 21,1%.

We observed a trend towards hypocellularity in MDS-RS-SLD (33.3%), MDS-RS-MLD (42.9%), and MDS-MLD (44.4%). A positive correlation, though not statistically significant, was also found between histopathological cellularity and the proportion of medullary blasts as assessed by cytomorphology.

When cellularity assessed by cytomorphology was used to find correlations, no statistically significant results were obtained, in accordance to the abovementioned results of the direct comparison of histopathological and cytomorphological cellularity assessment.

#### Erythropoiesis

The proportion of erythropoiesis did not correlate well between cytomorphological and histopathological review. Although histopathology showed superiority regarding overall cellularity assessment, only erythropoiesis diagnosed by cytomorphology showed a statistically significant correlation with cellularity (p=0.019). When assessed by histopathology, there was only a trend towards increased erythropoiesis in hypercellular marrows.

Neither WHO subtype nor medullary blast percentage correlated with the proportion of erythropoiesis in the marrow, irrespective of assessment by histopathology or cytomorphology.

The degree of dysmegakaryopoiesis, on the other hand, showed a trend towards positive correlation with the proportion of erythroid cells, when assessed by histopathology for both attributes. There was no patient with expanded erythropoiesis who did not demonstrate signs of dysmegakaryopoiesis.

#### WHO diagnosis

As shown in **Table 6**, we compared the histopathological and cytomorphological diagnoses. There was no case where histopathology did not confirm the diagnosis of MDS. 48% of MDS diagnoses were identical according to WHO type. However, in 56 cases (44%), the WHO type diagnosed by histopathology differed from the WHO type diagnosed by cytomorphology. The main reason was discordant estimation of medullary blast count. 32 patients were diagnosed with at least 5% medullary blasts by cytomorphology (MDS-EB1, MDS-EB2 and RAEB-T), while histopathology reported a normal blast count. Overestimation of blast count by histopathology occurred in 5 cases. In 8 cases, multi- versus unilineage dysplasia was identified as the discrepancy (MDS-SLD versus MDS-MLD, with or without ring sideroblasts). There were no cases where histopathology failed to diagnose CMML, but correlation regarding the distribution among CMML 0, I or II was week, reflecting the tendency of histopathology to underestimate the blast count.

**Table 6:** WHO diagnoses, histology vs cytology (χ^2 = ^345.4, p<0.00005).

	Histology									
	SLD	SLD RS	MLD	MLD-RS	EB1	EB2	AML MRC	CMML1	CMML2	Total
Cytology										
SLD	11	0	0	0	0	1	0	0	0	12
SLD-RS	0	2	0	1	0	0	0	0	0	3
MLD	4	0	7	5	1	1	0	0	0	18
MLD-RS	2	0	0	4	0	1	0	0	0	7
EB1	5	0	4	0	5	1	0	0	0	15
EB2	7	0	3	0	9	6	0	0	0	25
AML MRC	2	0	1	0	6	3	6	0	0	18
CMML1	0	0	0	0	0	0	0	20	1	21
CMML2	0	0	0	0	0	0	0	8	1	9

Red indicates strongly different findings between histopathology and cytomorphology, brown indicates more slightly different assessments (e.g. IB1 vs IB2).

#### Fibrosis

Evaluating the degree of fibrosis in a bone marrow specimen up to this day remains the preserve of histopathology. A positive correlation was found in our cohort between bone marrow cellularity and the degree of fibrosis. A high degree of fibrosis was predominantly observed in patients with hypercellularity (89.5% of fibrotic cases were hypercellular by histopathology). Similarly, a higher number of patients with a high degree of fibrosis was found in the high-risk subgroups of WHO 2016, namely MDS-EB2 (26.3%), RAEB-T (10.5%) and CMML I/II (31.6%). The same was true when the degree of fibrosis was compared with the percentage of medullary blast count, assessed by cytomorphology (**Table 7**).

**Table 7:** Degree of fibrosis vs medullary blast count by cytology (χ^2 = ^3.459, p= 0.063).

		Medullary blast count by cytology	
Degree of myelofibrosis			
	**0-4%**	**>4%**	**total**
**Grade 0-1**	45 (42%)	62 (58%)	107
**Grade 2-3**	4 (20%)	16 (80%)	20

The positive correlation between cellularity and fibrosis may appear counterintuitive, and the result should be interpreted with caution, due to the low number of patients with a high degree of fibrosis (n=20). However, a proportion of higher-risk MDS cases with elevated cellularity and blast counts may indeed have a tendency for fibrosis, which may have been underestimated so far.

#### Influence of histopathological and cytomorphological findings on overall prognosis

When the entire patient cohort was regrouped according to the blast count assessed by cytomorphology, the blast count showed a trend towards influencing median overall survival, especially in the patient groups with >5% blasts. The lack of statistical significance (p=0.128) is most likely attributable to the small size of the cohort. Regrouping based on histopathological assessment of blast count did not separate the cohort into subgroups with statistically significant different overall survival.

The presence of dysmegakaryopoiesis, identified on histopathology, did not show any prognostic impact, irrespective of the degree of dysmegakaryopoiesis.

Cellularity assessed by histopathology separated the cohort into three groups with different median survival times. A hypercellular marrow was associated with the worst outcome, even though statistical significance was not reached.

The proportion of erythropoiesis, again assessed by histopathology, seemed to influence overall survival when the patient cohort was divided into 5 groups (0-20%, 21-40%, 41-60%, 61-80%, >80%). Patients with 20-40% erythropoiesis showed a trend for the best overall survival. On cytomorphology, this effect had not been detectable. This might reflect the superiority of histopathological assessment already seen with regard to the overall cellularity.

**Figure 1:**
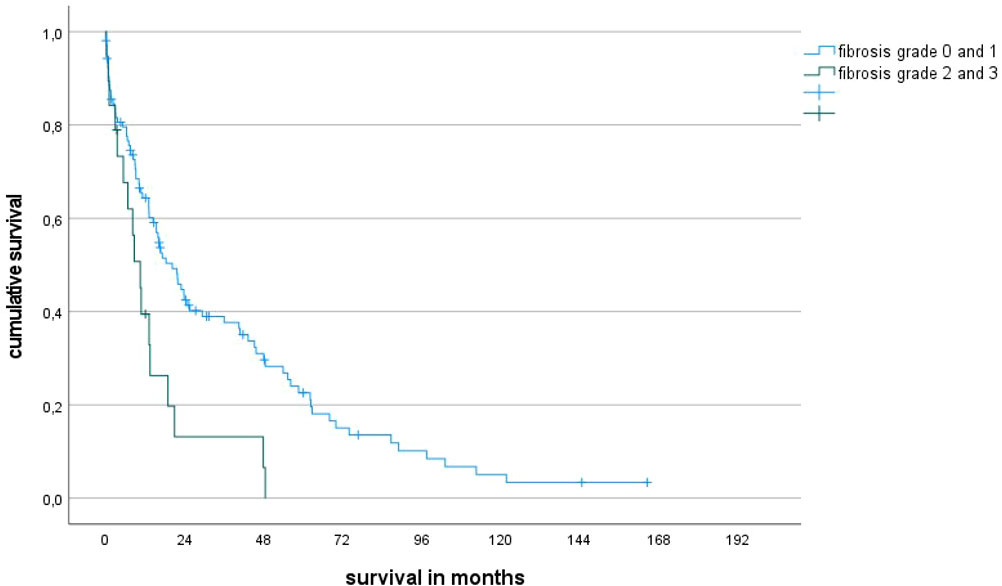
Overall survival rates of patients with fibrosis grade 2-3 vs 0-1 (median survival 10 ms vs 20 ms, p=0.004).

Presence of a high degree of fibrosis as assessed by histopathology translates into an inferior median survival as the degree of fibrosis separates the cohort into different subgroups with a statistically significant prognosis. Based on the degree of fibrosis, the entire patient cohort could be divided into two groups (no or mild signs of fibrosis versus high or very high degree of fibrosis) that showed a statistically significant difference in prognosis (**Figure 1**). The prognostic impact of fibrosis was also visible in WHO 2016 low-risk subgroups with a blast count <10%. The importance of fibrosis is reflected by the latest WHO classification for MDS, which now includes MDS with myelofibrosis (MDS-f).

## Discussion

Although cytomorphological examination of bone marrow aspirates, focusing on dysplastic features and blast counts, represents the gold standard for MDS diagnosis, additional information can be gained through histopathological evaluation of trephine biopsies (1, 2, 14). We compared cytomorphological and histopathological bone marrow analyses under well standardized conditions with regard to the congruence of the MDS diagnoses, typical MDS features such as dysplastic criteria and on the other hand features between both methods that are rather deemed central histopathological features (cellularity, fibrosis). All histopathological evaluations were done by the same expert pathologist (SB), and all cytomorphological diagnoses were made by the same expert hematologist (UG).

Based on the analysis of 128 MDS patients with regard to both cytomorphological and histopathological assessment of the bone marrow we could show that:

a) Histopathology reliably recognizes the presence of MDS but underestimates the blast count and can therefore not correctly classify MDS patients according to WHO classification.
b) On the other hand, cytomorphology cannot reliably assess bone marrow cellularity and tends to overestimate it (3–6). As the new WHO 2022 classification includes hypocellular MDS (MDS-h) (16), hematologists are now obliged to include a bone marrow trephine biopsy in their diagnostic workup of MDS.
c) When comparing the assigned MDS subtype within our cohort by WHO 2016 with the most recent WHO 2022 classification, it shows that 5,5% of patients are reassigned to the newly created subgroup of MDS-f and is constituted by patients with a former subtype of EB-1 and EB-2. Acknowledging this subtype with high prognostic significance, even in lower blast MDS as described above, is only possible when performing histopathologic assessment. All patients with hypocellular MDS, by definition, are patients of the former low blast subgroups MDS-SLD and –MLD. As in our cohort no patient apart from SF3B1 was included with MDS-defining cytogenetic or molecular aberrations such as del(5q) or biallelic TP53 there was no assignment to the respective subgroups by WHO 2022. As we classified patients according to the WHO classification, there is no shift in additional cases classified as AML as we only have cases with IB1 order IB2 and no additional AML cases as proposed in the ICC using a cut-off of more than 10%.
d) Fibrosis is an important prognostic factor in MDS that can only be assessed by histopathology. We found that fibrosis shows a positive correlation with bone marrow cellularity and the medullary blast count. The new WHO 2022 classification pays tribute to the importance of fibrosis by including MDS with myelofibrosis (MDS-f) as one of the MDS subtypes (16).
e) Dysmegakaryopoiesis seems to be another feature that is properly assessed by histopathology. We found that the degree of dysmegakaryopoiesis correlates with cellularity and unfavorable WHO categories and MDS risk groups.

We consider histopathology a valuable supplement in the diagnostic workup of MDS. Superiority to a cytomorphologically assessed MDS diagnosis could not be demonstrated, mainly due to its inability to assess subtle morphological features at the level of individual cells except megakaryocytes. Nevertheless, histopathology offers complementary information regarding fibrosis and cellularity that contributes substantially to prognostic assessment. The importance of histopathology is reflected in the new WHO 2022 classification, which includes MDS types (MDS-h and MDS-f) that require histopathology for proper assessment (17).

## Data availability statement

The raw data supporting the conclusions of this article will be made available by the authors, without undue reservation.

## Ethics statement

The studies involving humans were approved by Ethikkommission der Medizinischen Fakultät der Heinrich Heine Universität Duesseldorf Votum 3973 Projekt MDS Register. The studies were conducted in accordance with the local legislation and institutional requirements. The human samples used in this study were acquired from a by- product of routine care or industry. Written informed consent for participation was not required from the participants or the participants’ legal guardians/next of kin in accordance with the national legislation and institutional requirements.

## Author contributions

KN: Conceptualization, Data curation, Formal analysis, Investigation, Methodology, Validation, Visualization, Writing – original draft, Writing – review & editing. CS: Data curation, Methodology, Validation, Writing – review & editing. RF: Data curation, Formal analysis, Methodology, Validation, Writing – review & editing. NG: Data curation, Methodology, Validation, Writing – original draft, Writing – review & editing. SD: Data curation, Methodology, Validation, Writing – review & editing. UG: Conceptualization, Data curation, Formal analysis, Investigation, Methodology, Validation, Writing – original draft, Writing – review & editing. SB: Conceptualization, Investigation, Methodology, Validation, Writing – review & editing.
